# Pharmacovigilance of nephrotoxic drugs in neonates: the Pottel method for acute kidney injury detection in ELBW neonates

**DOI:** 10.1007/s00467-024-06335-3

**Published:** 2024-03-25

**Authors:** Mathilde Dumoulin, Hans Pottel, Djalila Mekahli, Annouschka Laenen, Anne Smits, Karel Allegaert

**Affiliations:** 1 Department of Paediatrics, Leuven University Hospitals, Louvain, Belgium; 2 Department of Public Health and Primary Care, KU Leuven Campus Kulak, Kortrijk, Belgium; 3 Department of Pediatric Nephrology, Leuven University Hospitals, Louvain, Belgium; 4 PKD Research Group, Department of Cellular and Molecular Medicine, KU Leuven, Louvain, Belgium; 5 Leuven Biostatistics and Statistical Bioinformatics Center (L-BioStat), KU Leuven, Louvain, Belgium; 6 Department of Development and Regeneration, KU Leuven, Herestraat 49, 3000 Louvain, Belgium; 7 Neonatal Intensive Care Unit, Leuven University Hospitals, Louvain, Belgium; 8 Department of Pharmaceutical and Pharmacological Sciences, KU Leuven, Louvain, Belgium; 9 Department of Hospital Pharmacy, Erasmus MC University Medical Center, Rotterdam, The Netherlands

**Keywords:** Extremely low birth weight (ELBW), Acute kidney injury (AKI), Serum creatinine, Biomarker, Pottel method, Pharmacovigilance, Ibuprofen

## Abstract

**Background:**

Extremely low birth weight (ELBW) neonates (birth weight ≤ 1000 g) are at high risk to develop drug-induced acute kidney injury (AKI). However, we lack a pragmatic detection tool to capture their time-dependent (patho)physiologic serum creatinine (Scr) patterns. Pottel et al. suggested rescaling Scr by dividing Scr with the mean Scr value of the age- and sex-specific reference population. We explored if this Pottel method can detect drug-related nephrotoxicity in ELBW neonates.

**Methods:**

A previously reported dataset on Scr changes in ELBW neonates exposed to ibuprofen, amikacin, or vancomycin was updated to calculate Pottel scores for every available Scr value in the first 28 postnatal days. We hereby used previously published postnatal age-specific 50^th^ centile values in an ELBW population. Linear mixed models were applied, analyzing Pottel scores as response variable and continuous time (day), drug exposure, and interaction thereof in the explanatory model.

**Results:**

Serum creatinine (*n* = 3231) observations in 201 ELBW neonates were collected. A statistically significant rise of Pottel scores was observed with ibuprofen starting from postnatal day 4. In addition, a cumulative effect of treatment with mean Pottel scores on day 0 of 1.020 and on day 3 during treatment of 1.106 (95% CI 1.068–1.145, *p* < 0.001) was observed, corrected for effect of antibiotics. Antibiotic administrations showed a small but statistically significant difference up to postnatal day 5.

**Conclusions:**

As rescaled Scr biomarker, the Pottel method showed a clear association with ibuprofen-exposed ELBW neonates, suggesting its applicability as a pragmatic bedside alternative tool to assess nephrotoxicity.

**Graphical abstract:**

**Figure:**
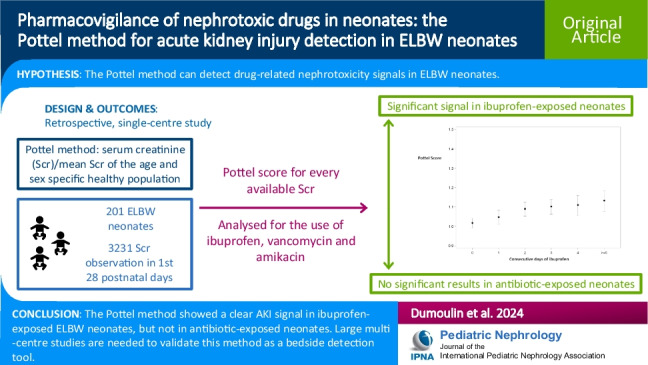
A higher resolution version of the Graphical abstract is available as Supplementary information

**Supplementary Information:**

The online version contains supplementary material available at 10.1007/s00467-024-06335-3.

## Introduction

Extremely low birth weight (ELBW) premature neonates (birth weight (BW) ≤ 1000 g) are born during active nephrogenesis [1, 2]. This population has a high risk to develop acute kidney injury (AKI) due to immature kidney physiology combined with several possible risk factors during their neonatal intensive care unit (NICU) stay, such as hemodynamic instability, late-onset sepsis (LOS), necrotizing enterocolitis (NEC), or persistent ductus arteriosus (PDA) [3–6]. These events often co-occur with nephrotoxic medication exposure, in up to 92% of ELBW neonates [6]. The recent Baby Nephrotoxic Injury Negated by Just-in-Time Action (BABY-NINJA) trial showed that identifying neonates at risk for nephrotoxic drug-induced AKI and implementing guidelines to reduce the exposure resulted in a significant decrease in AKI prevalence and intensity [5]. Consequently, early and consistent precision pharmacovigilance holds the potential to reduce or prevent AKI in ELBW neonates. However, this needs a pragmatic bedside tool.

The most currently applied definition for AKI in ELBW neonates is the neonatal modification of the KDIGO (Kidney Disease: Improving Global Outcomes, mKDIGO) definition for adults and children [7]. The mKDIGO is a grading system, based on different rises of serum creatinine (Scr), where a rise of 0.3 mg/dL or ≥ 50% compared to the previous value defines stage 1 AKI [7].

However, the pattern of physiologic Scr changes in ELBW neonates is characterized by an initial progressive Scr increase in the first days peaking on days 3–4 and a subsequent slow decrease, with interindividual variability due to maturational and non-maturational factors like drugs or fluid therapy [8, 9]. Consequently, the mKDIGO has limitations as it assumes a steady state and will not detect the absence of an appropriate, expected decline of Scr in the second part of the first week of life and beyond. It also requires consecutive Scr measurements.

The idea to explore assay-specific mean and centile Scr values to describe postnatal trends was introduced, as described by Pottel et al. [10, 11]. They suggested to normalize Scr by dividing Scr by the mean Scr value of the appropriate age- and sex-specific healthy population, rescaling it to a kidney biomarker with a normal distribution around the mean of 1 [10]. However, this effort was done from age 2 years onwards.

In previous work on Scr patterns in ELBW neonates, a modest decrease of creatinine clearance and a shift of Scr of about 1 standard deviation (SD) in ibuprofen-exposed neonates were documented, as well as a minor increase of Scr when treated with amikacin and/or vancomycin [11–13].

In the present study, we investigated whether the Pottel method also enables detection of drug-related nephrotoxicity in this dataset. This alternative approach would make detection more feasible bedside, is based on a single observation, and mitigates the time-dependent Scr pattern.

## Methods

### Study population and clinical characteristics

A previously reported dataset, used to define Scr trends and drug toxicity in ELBW neonates admitted to the NICU of the University Hospitals Leuven in two periods (July 2007 to August 2011 and June 2015 to March 2017), was reused [9, 12, 13]. This dataset contains retrospectively collected data on demographics, relevant clinical data (e.g., mode of delivery, exposure to inotropics), and days of treatment with ibuprofen, amikacin, or vancomycin and the available Scr for the first 42 days of life [9]. The Leuven NICU uses ibuprofen for the pharmacological PDA closure, whereas the combination of amikacin and vancomycin is the regimen for suspected LOS (late-onset sepsis, > 72-h postnatal age). Amikacin is also used as co-treatment with amoxicillin for early-onset sepsis (< 72 h) and with piperacillin-tazobactam to treat necrotizing enterocolitis.

Serum creatinine was analyzed enzymatically (Roche Diagnostics, Mannheim, Germany) during the study period, and all measurements were isotope dilution mass spectrometry (IDMS) traceable [9].

**Fig. 1:**
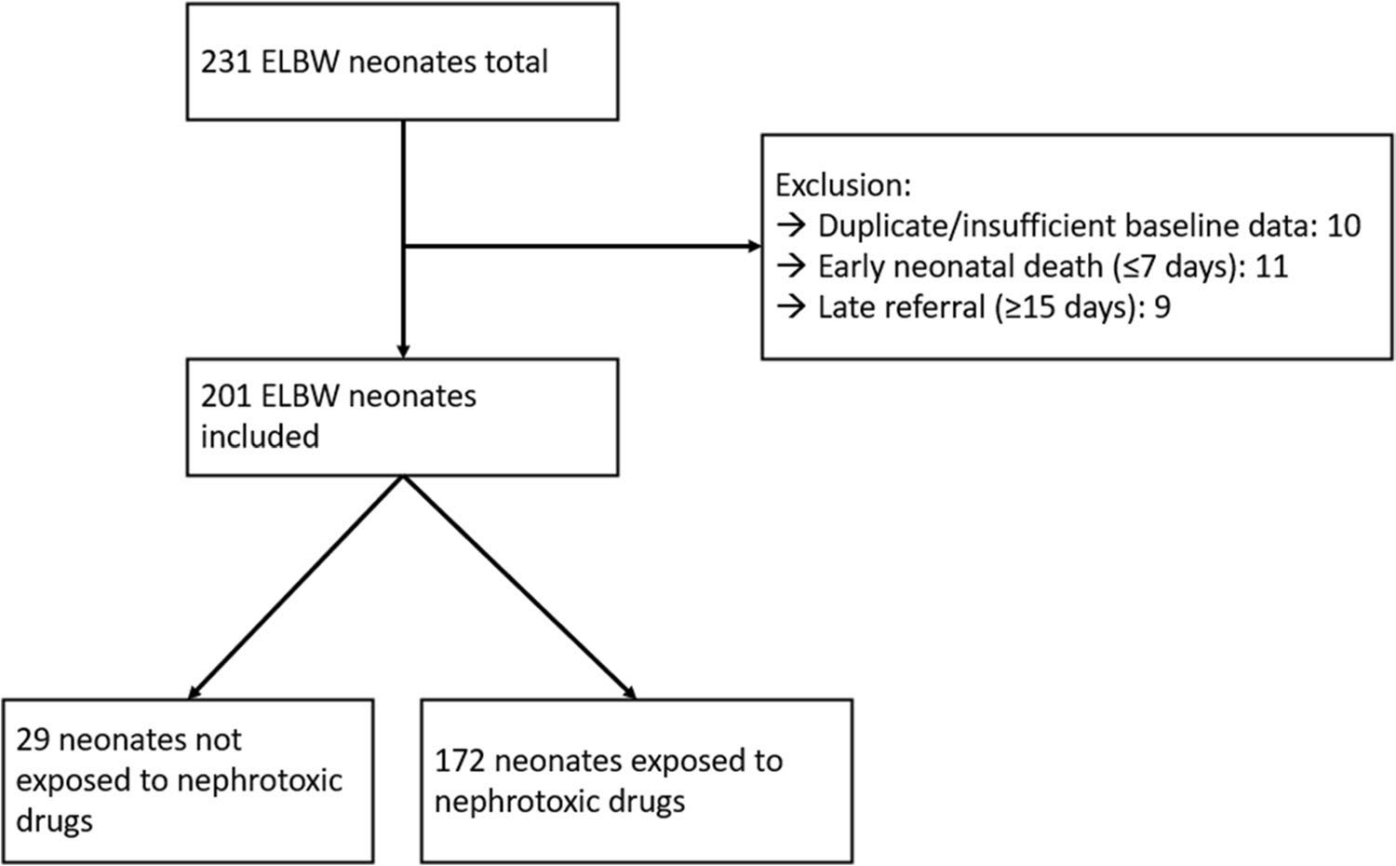
Selection of study population with respective exclusions. Nephrotoxic drugs include ibuprofen, amikacin, and vancomycin. *ELBW*, extremely low birth weight

We restricted the dataset to observations obtained in the first 28 days after birth. After review of the available data, 30 patients were excluded due to insufficient baseline data, early neonatal death (< 7 days after birth), late referral (≥ postnatal age (PNA) day 15), or duplication. This resulted in 3231 Scr observations in 201 patients (Fig. 1 and Figure S1). Pottel scores for every available Scr were calculated based on 50^th^ centile values per postnatal day in ELBW neonates (Supplementary Table 1, column 2), as previously published [11]. Ethics approval was provided (S63405, Ethics Committee Research UZ/KU Leuven).

### Statistical methods

Patient characteristics were described by mean and SD or frequencies with percentages (Table 1). Descriptive summary statistics of the Pottel score were calculated, including mean and SD, based on values collected from days without influence of ibuprofen or antibiotics (i.e., no administration of any of these drugs the day before Scr measurement was performed).

**Table 1:** Demographic data presented as mean (standard deviation) or as number (%). Data on use of prenatal steroids, mode of delivery, and antibiotic exposure are calculated in 197, 199, and 200 patients, respectively, due to missing data. *obs* observations

Patient characteristics	Number
ELBW neonates (obs)	201 (3231)
Gestational age (weeks)	27 (2)
Birth weight (grams)	808 (135)
Sex	
Female	107 (53.2%)
Male	94 (46.8%)
Mode of delivery	
Vaginal	63 (31.7%)
Caesarean	136 (68.3%)
Lung maturation (yes)	171 (86.8%)
Late neonatal death (yes)	17 (8.5%)
Ibuprofen exposure (yes)	124 (61.7%)
Amikacin exposure (yes)	151 (75.5%)
Vancomycin exposure (yes)	152 (76%)
Amikacin exposure without vancomycin exposure (yes)	10 (5.0%)
Vancomycin exposure without amikacin exposure (yes)	11 (5.5%)
Amikacin and vancomycin exposure (yes)	141 (70.5%)
No ibuprofen or antibiotic exposure	29 (14.4%)

To evaluate the effect of ibuprofen and antibiotics (amikacin and/or vancomycin) on the Pottel score and to determine whether the score showed a significant association, linear mixed models were used, with the Pottel score as response variable and continuous time (PNA, expressed in days), pharmacotherapy (ibuprofen, amikacin, and/or vancomycin, yes/no), and the interaction thereof in the explanatory models. Random intercept and slope in time were modeled to account for the longitudinal data structure. In addition, the combined effects of ibuprofen and antibiotics were used in multivariable models to account for possible confounding effects by estimating the effect of ibuprofen corrected for antibiotics, and vice versa.

As the effect of pharmacotherapy is expected to be seen with a 1-day delay (“lag” time), treatment was modeled as binary variable, with value 1 if the respective treatment was administered on the day before outcome evaluation and value 0 if otherwise. Log transformation was applied to the time variable to deal with non-linear trends over time, as this resulted in a better model fit (lower Akaike information criterion) compared to cubic spline models.

Results were presented graphically, presenting estimated Pottel score over time with 95% confidence bands in the presence or absence of drug exposure.

To evaluate the cumulative time effect of ibuprofen or antibiotic administration on the Pottel score, we used similar linear mixed models for data analysis, modeling the Pottel score as an effect of the number of consecutive days of drug administration.

Analyses were performed using SAS software (version 9.4, SAS Institute Inc, Cary, NC, USA) for Windows. A *p*-value of < 0.05 was considered statistically significant.

## Results

### Study population

Data from 201 ELBW neonates with a mean BW of 808 (range 370–1000, interquartile range IQR 710–900) grams and a mean gestational age (GA) of 27 (23–34, IQR 25–28) weeks were available. Most neonates (*n* = 171, 86.8%) received prenatal lung maturation (maternal steroids), and 136 neonates (68.3%) were born following caesarean delivery. Late neonatal death (PNA day ≥ 7) occurred in 17 patients (8.5%) (Table 1).

During neonatal life (PNA days 1–28), 124 neonates (61.7%) received ibuprofen, 151 (75.5%) amikacin, and 152 (76%) vancomycin. Ibuprofen was administered for a median of 3 (range 1–14) days, amikacin for 6 (range 1–17) days, and vancomycin for 7 (range 1–21) days. Only 29 (14.4%) patients never received any of these nephrotoxic drugs (Table 1). There were missing data for four patients concerning use of prenatal steroids, for two patients concerning mode of delivery, and for one patient concerning administration of any antibiotic respectively, which were excluded for their respective analyses. The number of available Scr observations for each day is provided in Supplementary Table 1, column 3, and the pattern in Figure S1.

### Pottel scores

To explore Pottel score distribution without impact of drug exposure, scores collected on days without drug administration on the previous days were used to obtain summary statistics. This analysis revealed a normal distribution with a mean of 1.01 (SD 0.257).

### Association with drug administration over postnatal age (Supplementary Table 2)

#### Ibuprofen

**Fig. 2:**
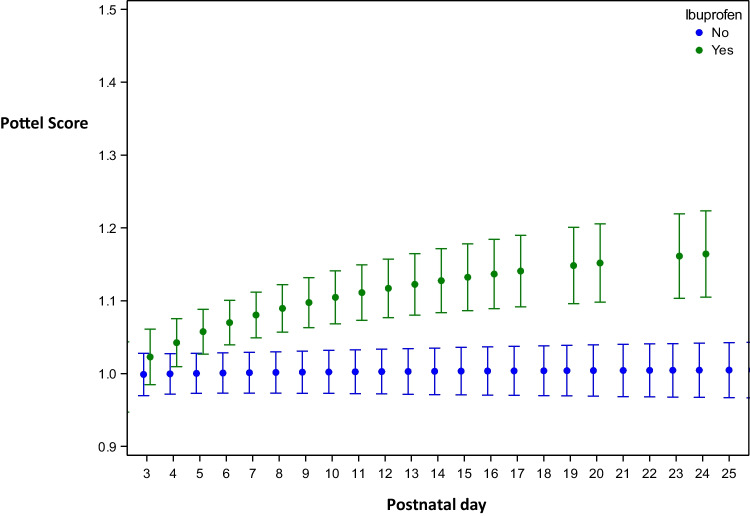
Mean (standard deviation) Pottel score per postnatal day (days 1–28) in function of administration of ibuprofen the day before or not, corrected for administration of amikacin and/or vancomycin. The estimated curves are presented within the range of observations: days on which (almost) no influence of ibuprofen was present (defined as administration to none or maximal one newborn on the day before) are not shown. Estimated means are presented with 95% confidence intervals

Pottel scores, calculated in function of ibuprofen exposure on the previous day, showed statistically significant differences starting from PNA day 4 with higher Pottel scores after exposure compared to no exposure (on PNA day 4 mean difference in Pottel score 0.040 [0.017; 0.063], *p* = 0.0005). The magnitude of differences increased with PNA, with larger confidence intervals reflecting more dropout cases, reflecting a sample size effect. Results of ibuprofen corrected for administration of antibiotics were comparable (Fig. 2).

#### Antibiotics

**Fig. 3:**
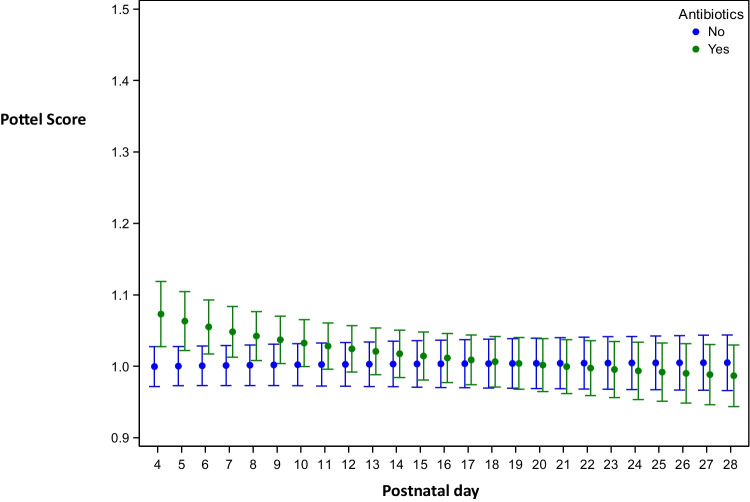
Both antibiotics (amikacin and vancomycin) were combined for this analysis. Mean (standard deviation) Pottel score per day (postnatal, days 1–28) in function of administration of antibiotics the day before or not, corrected for administration of ibuprofen. The estimated curves are presented within the range of observations: days on which (almost) no influence of antibiotics was present (as there was administration to none or one child on the day before) are not shown. Estimated means are presented with 95% confidence intervals

Effects for amikacin alone, vancomycin alone, and a combination of both were calculated, with comparable results for these three analyses. Antibiotic administration resulted in a small but statistically significant difference in Pottel scores up to PNA day 5 (on PNA day 5 for amikacin mean difference 0.070 [0.035; 0.106], *p* = 0.0001; for vancomycin mean difference 0.080 [0.045; 0.114] *p* ≤ 0.0001; for antibiotics combined mean difference 0.069 [0.036; 0.102], *p* ≤ 0.0001). In contrast, starting from PNA day 25, we observed a statistically significant difference resulting in a lower Pottel score with amikacin or vancomycin administration alone, but not with both combined (on PNA day 25 for amikacin mean difference − 0.030 [− 0.054; − 0.006], *p* = 0.0159; for vancomycin mean difference − 0.034 [− 0.058; − 0.010], *p* = 0.0057; for antibiotics combined mean difference − 0.022 [− 0.044; 0.001], *p* = 0.0610). When correcting for ibuprofen, again, similar results were obtained with statistically significant differences up to PNA day 5 (Fig. 3).

### Cumulative effect during treatment over consecutive days (Supplementary Table 3)

#### Ibuprofen

**Fig. 4:**
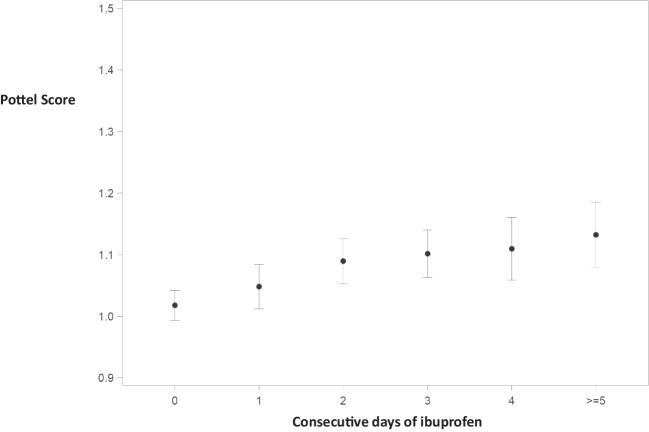
Cumulative impact of consecutive days of administration of ibuprofen, corrected for amikacin and/or vancomycin on the Pottel score. Estimated means are presented with 95% confidence intervals

A clear cumulative effect of ibuprofen on the Pottel score was observed, with significant differences starting from day 1 after administration (mean Pottel score on day 0 1.018 [0.994; 1.042], on day 1 of administration 1.048 [1.012; 1.085], *p* = 0.0393). A significant further rise of scores in the following days of exposure was seen, with a subsequent flattening of the mean scores (mean Pottel score on day 3 1.101 [1.063; 1.140], *p* ≤ 0.0001 and on day ≥ 5 1.132 [1.079; 1.185], *p* ≤ 0.0001). When corrected for antibiotics there is a similar trend (mean Pottel score on day 0 1.020 [0.995; 1.044], on day 3 1.106 [1.068; 1.145], *p* ≤ 0.0001, and on day ≥ 5 1.135 [1.082; 1.187], *p* ≤ 0.0001) (Fig. 4).

#### Antibiotics

**Fig. 5:**
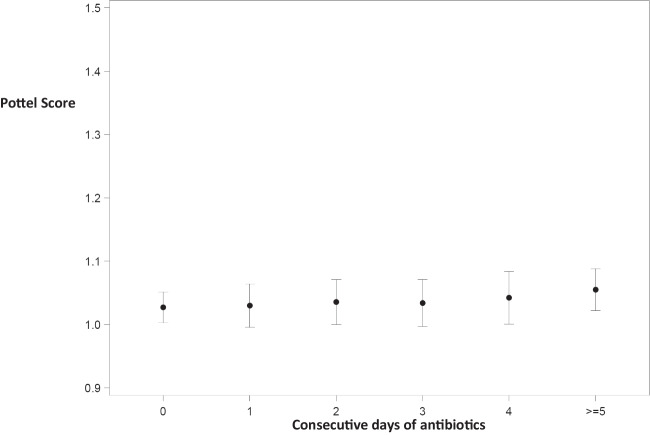
Cumulative impact of consecutive days of administration of amikacin and/or vancomycin, corrected for ibuprofen. Estimated means are presented with 95% confidence intervals

A small statistically significant difference was observed only on ≥ 5 days of treatment (mean Pottel score on day 0 1.027 [1.002; 1.052], on day 3 of administration 1.034 [0.997; 1.071], *p* = 0.6399, and on day ≥ 5 of administration 1.055 [1.022; 1.088], *p* = 0.0194), with similar results when corrected for ibuprofen (mean Pottel score on day 0 1.024 [0.999; 1.048] and on day ≥ 5 of administration 1.060 [1.027; 1.093], *p* = 0.0021) (Fig. 5).

## Discussion

In this retrospective study, the applicability of the Pottel method—a rescaled Scr marker by dividing Scr with the mean Scr value of the age- and sex-specific healthy population (Qcrea)—to detect drug-induced nephrotoxicity in ELBW neonates was assessed.

AKI is common in ELBW neonates, with incidences up to 60% [3, 14–16]. ELBW neonates already have a decreased kidney reserve due to their disrupted kidney development, with evidence showing smaller kidney size and abnormal kidney-related outcomes in childhood compared to healthy controls [1, 2, 17]. Experiencing neonatal AKI likely further imposes an increased risk of long-term kidney dysfunction [17, 18]. These patients are often exposed to nephrotoxic drugs, which co-occurs with several known risk factors such as hemodynamic instability, LOS, NEC, or PDA [3–6]. Consequently, timely detection of AKI during the use of nephrotoxic drugs could reduce AKI rates in ELBW neonates and improve long-term kidney outcomes. However, accurate detection of AKI remains challenging. Several definitions are available, such as the predominantly used mKDIGO, the pRIFLE (pediatric Risk, Injury, Failure, Loss, End-stage kidney disease), or AKIN (Acute Kidney Injury Network), all showing similar AKI incidences in ELBW neonates [16]. These definitions are all based on defined changes in urine output (UOP) or Scr, assuming a steady-state situation and needing at least two observations. ELBW neonates, however, exhibit a physiologic postnatal Scr rise from the day of birth (day 1) to approximately day 3 with a subsequent decline, delayed more with increasing immaturity [8]. The Scr rise over the first days of life noted in ELBW in many instances crosses the thresholds to diagnose stage 1 AKI by the modified, neonatal KDIGO definition (Figure S1). However, in these neonates, this may be consistent with a normal, physiologic phenomenon rather than a pathophysiologic state. The use of current definitions is therefore limited in preterm neonates due to their time-dependent Scr physiology as well as challenges in accurately measuring UOP [19].

This scenario serves as an example of the utility of a rescaled Scr marker, such as the Pottel method, which may be better for detection of truly abnormal Scr than the present KDIGO criteria in circumstances such as these. Pottel et al. analyzed the use of their rescaled Scr marker with a multicohort study in a population > 2 years of age demonstrating a distribution of Scr/Qcrea around 1, with the 2.5th and 97.5th percentile at 0.67 and 1.33, respectively, showing specificity and sensitivity for impaired kidney function close to 90% over all age groups when using 1.33 as the cut-off value [10, 20].

We hypothesized that the Pottel method could be useful to explore (drug-related) nephrotoxicity in ELBW cases. An existing cohort, previously used to describe Scr patterns in ELBW neonates during ibuprofen, amikacin, or vancomycin exposure, was used as we knew this cohort showed an association between drug exposure and kidney impairment [12, 13].

In ibuprofen-exposed neonates, a clear association was observed with a statistically significant increase in Pottel scores during ibuprofen treatment starting from PNA day 4. There was a progressive difference in scores, increasing with advancing age, suggesting a more robust nephrotoxic AKI effect, confirming previous analyses [11, 12]. Exploring the cumulative effect during ibuprofen exposure revealed a rising Pottel score until day 3 of exposure followed by a relatively stable value thereafter. This emphasizes the importance of well-timed assessments during drug treatment before drawing definitive conclusions about potential kidney damage. Early measurements might not fully capture the full nephrotoxic effect.

In contrast, we could not show a significant association beyond PNA day 5 when evaluating the Pottel score during the use of amikacin and/or vancomycin. This seems to reflect a less pronounced nephrotoxic effect of these antibiotics in our study population. In our prior analysis, there was also a more modest change in Scr dynamics during amikacin and vancomycin treatment compared to ibuprofen [13]. Unexpectedly, we even noticed a decreased Pottel score in the latter half of the first month of life. As this study was explorative, we describe this finding, but fail to explain it. Among other causes, this could be due to less robust *Q* reference values in the third and fourth week in the currently available dataset as one of the limitations of the current analysis.

There are obvious limitations. Likely most relevant, the p50 values used for calculating the Pottel scores were derived from the same cohort in a previous analysis, influencing statistical outcomes [11]. However, our main intention was to introduce the concept. We do not claim that the p50 Scr values applied (Supplementary Table 1, column 2) to convert absolute Scr values to Pottel scores are ready reference values as they were derived from a single-center, small dataset (Table 1) [11]. Our primary goal was to illustrate the potential strength of this approach, as Scr displays extensive time-dependent variability in the first weeks of postnatal age, not yet at steady state. We do not claim that this approach is already a robust substitute for the currently used AKI definitions. A next obvious step would be to determine p50 values for Scr for a diversity of subpopulations commonly admitted to NICUs and to validate the approach used. Preferably, these datasets are generated from different units to reflect diversity in practices and settings.

We did not account for prolonged medication effects after cessation of treatment, nor did we study the impact of other drugs as we did not have sufficient granularity in our current dataset. The AWAKEN study showed that diuretics, vasopressors, and methylxanthines were associated with a lower risk for early onset AKI (postnatal days 2–7), whereas some medications were associated with increased odds for late AKI (> 7 days after birth) in neonates [4, 21]. The impact on the Pottel score could be different based on the type and duration of drug exposure. We neither accounted for other known variables in ELBW neonates that may have impacted kidney health, e.g., prenatal factors such as maternal hypertension or certain drug exposure or postnatal factors such as hypotension or elevated mean airway pressure, as we did not have sufficient data available in our retrospective dataset [22].

Considering these limitations, our findings underscore the potential value of the Pottel method in Scr-based bedside pharmacovigilance for ELBW neonates as an alternative technique. This approach enables bedside assessment based on a single Scr value and captures the time-dependent pattern. In addition, it could provide a way to standardize AKI definitions in clinical trials, improving comparability in this population. It is important to note that the established upper limit of 1.33 (for chronic kidney disease and in children > 2 years) does not seem to be applicable in ELBW neonates. Our initial findings hint towards a considerably lower threshold. Scr remains a widely recognized and measured biomarker, and the application of the Pottel method moderates certain inherent limitations linked to its use in this population. Additional steps, with comprehensive observational large-scale multicentre studies, are needed to further explore and develop this approach, to ascertain solid p50 values, to make the current findings more generalizable, and to compare this tool to the currently used AKI definitions.

## Conclusion

As rescaled Scr biomarker, the Pottel method showed a clear association with ibuprofen-exposed ELBW neonates, suggesting its applicability as a pragmatic bedside alternative tool to assess nephrotoxicity. We suggest further exploration and development of this alternative approach.

### Supplementary Information

Below is the link to the electronic supplementary material.

Graphical Abstract (PPTX 457 KB)

Supplementary file2 (DOCX 848 KB)

## Data Availability

The corresponding author can be contacted. Data availability will be considered, if based on a reasonable study proposal.
