# Supplementary material for: Pharmacovigilance of nephrotoxic drugs in neonates: the Pottel method for acute kidney injury detection in ELBW neonates
Source: Pediatr Nephrol. 2024 Mar 25;39(8):2525–32. doi: 10.1007/s00467-024-06335-3 (PMC11199258; doi:10.1007/s00467-024-06335-3)
Supplement: Supplementary file 1 — Graphical Abstract (PPTX 457 KB) [file 467_2024_6335_MOESM1_ESM.pptx]

## Slide 1
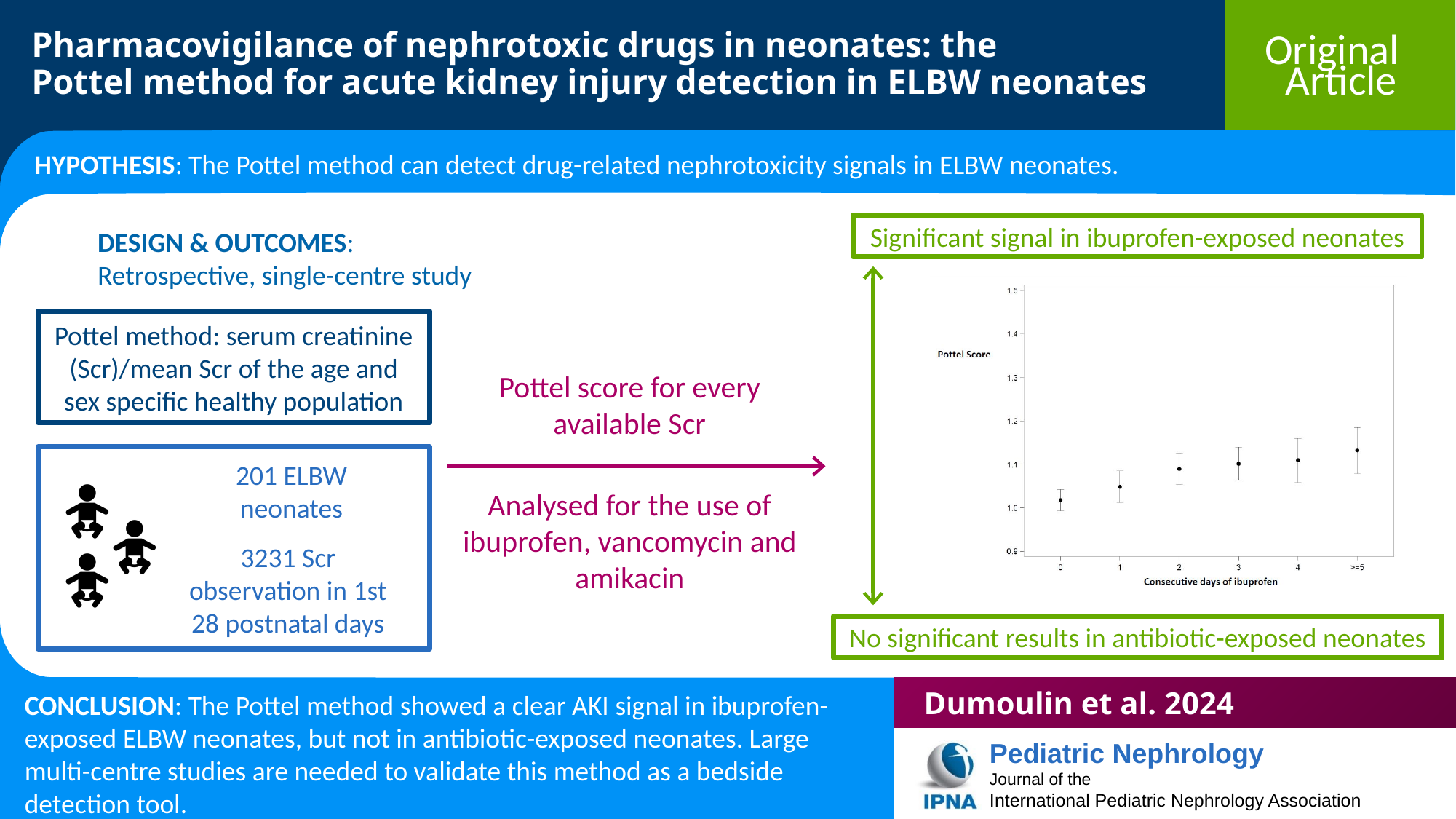

Pharmacovigilance of nephrotoxic drugs in neonates: the
Pottel method for acute kidney injury detection in ELBW neonates
HYPOTHESIS: The Pottel method can detect drug-related nephrotoxicity signals in ELBW neonates.
Significant signal in ibuprofen-exposed neonates
DESIGN & OUTCOMES:
Retrospective, single-centre study
Pottel method: serum creatinine (Scr)/mean Scr of the age and sex specific healthy population
Pottel score for every available Scr
201 ELBW neonates
Analysed for the use of ibuprofen, vancomycin and amikacin
3231 Scr observation in 1st 28 postnatal days
No significant results in antibiotic-exposed neonates
Dumoulin et al. 2024
CONCLUSION: The Pottel method showed a clear AKI signal in ibuprofen-exposed ELBW neonates, but not in antibiotic-exposed neonates. Large multi-centre studies are needed to validate this method as a bedside detection tool.
