# Supplementary material for: Pharmacovigilance of nephrotoxic drugs in neonates: the Pottel method for acute kidney injury detection in ELBW neonates
Source: Pediatr Nephrol. 2024 Mar 25;39(8):2525–32. doi: 10.1007/s00467-024-06335-3 (PMC11199258; doi:10.1007/s00467-024-06335-3)
Supplement: Supplementary file 2 — Supplementary file2 (DOCX 848 KB) [file 467_2024_6335_MOESM2_ESM.docx]

**Supplementary Figure 1**

Pattern of all available serum creatinine observations (n = 3231) in 201 Extremely low birth weight neonates retained in the current analysis.


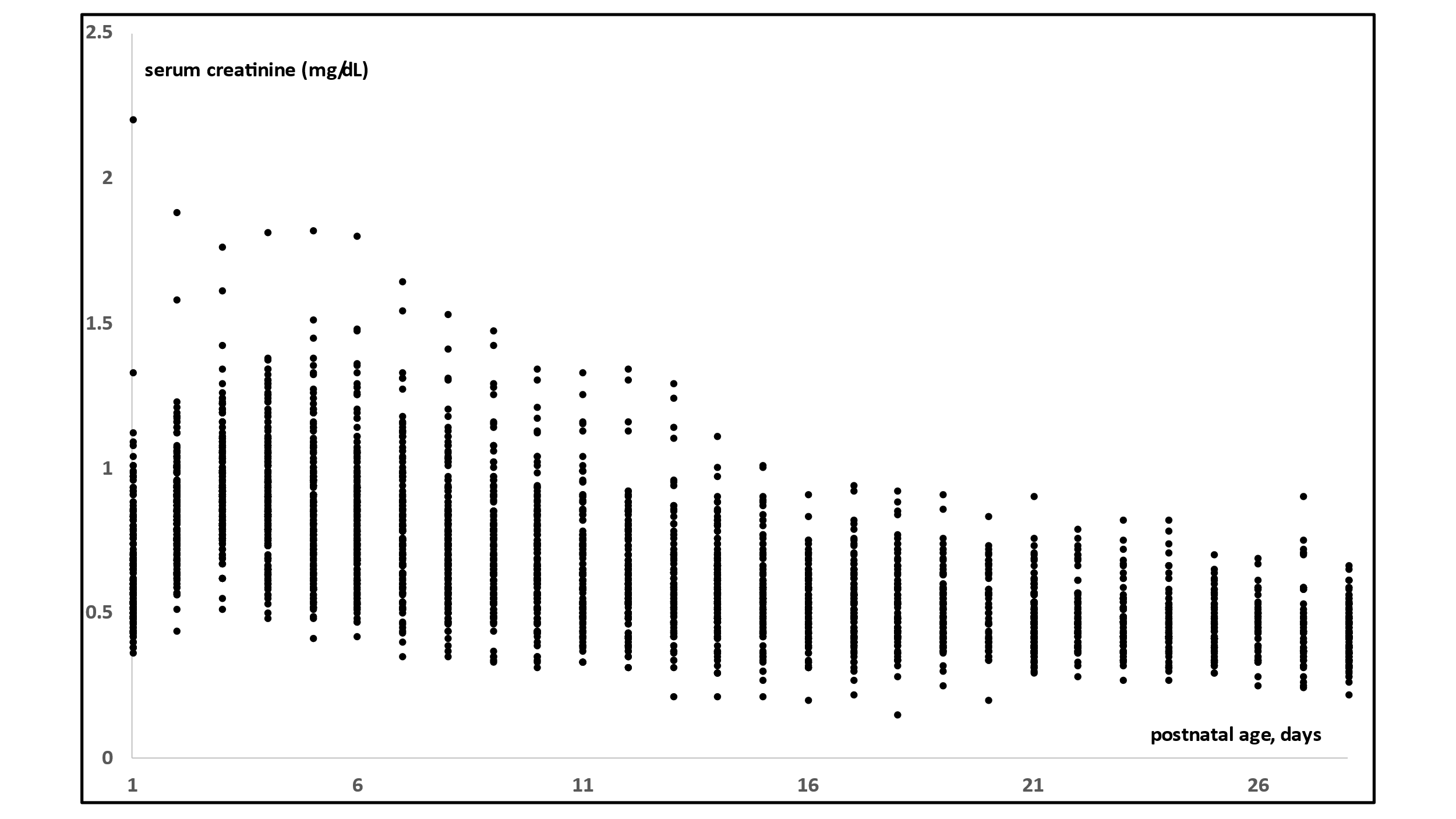


**Supplementary Table 1**

Overview on the 50th centile serum creatinine values, derived from the same cohort analysed in a previous paper, used to calculate the Pottel scores [11]. The 3^rd^ column provided an overview of the number of available observations of each day.

| **postnatal age (day)** | **50th centile (mg/dl)** [11] | **number of observations** |
| --- | --- | --- |
| day 1 | 0.60 | 194 |
| day 2 | 0.86 | 178 |
| day 3 | 0.91 | 194 |
| day 4 | 0.88 | 190 |
| day 5 | 0.84 | 177 |
| day 6 | 0.80 | 168 |
| day 7 | 0.75 | 142 |
| day 8 | 0.74 | 156 |
| day 9 | 0.70 | 140 |
| day 10 | 0.65 | 128 |
| day 11 | 0.64 | 117 |
| day 12 | 0.60 | 111 |
| day 13 | 0.59 | 92 |
| day 14 | 0.57 | 131 |
| day 15 | 0.55 | 98 |
| day 16 | 0.52 | 95 |
| day 17 | 0.51 | 97 |
| day 18 | 0.49 | 76 |
| day 19 | 0.49 | 82 |
| day 20 | 0.50 | 57 |
| day 21 | 0.47 | 120 |
| day 22 | 0.46 | 68 |
| day 23 | 0.47 | 63 |
| day 24 | 0.47 | 83 |
| day 25 | 0.43 | 62 |
| day 26 | 0.45 | 50 |
| day 27 | 0.44 | 53 |
| day 28 | 0.42 | 108 |

**Supplementary Table 2**

The association of the Pottel score with drug administration by postnatal age. A mean difference > (<) 0 implies a higher (lower) Pottel score under influence of the respective drug. The selected days were based on the characteristics of the dataset. CI: confidence interval.

| **Drug** | **Postnatal day** | **Mean difference in Pottel score (95% CI)** | **P-value** |
| --- | --- | --- | --- |
| Ibuprofen | 3 | 0.020 (-0.010;0.050) | 0.1938 |
|  | 4 | 0.040 (0.017;0.063) | 0.0005 |
|  | 5 | 0.056 (0.037;0.074) | <.0001 |
| Ibuprofen, corrected for amikacin or vancomycin | 3 | 0.024 (-0.006;0.054) | 0.1177 |
|  | 4 | 0.043 (0.020;0.065) | 0.0002 |
|  | 5 | 0.057 (0.039;0.076) | <.0001 |
| Amikacin | 5 | 0.070 (0.035;0.106) | 0.0001 |
|  | 15 | 0.002 (-0.013;0.017) | 0.7823 |
|  | 25 | -0.030 (-0.054;-0.006) | 0.0159 |
| Vancomycin | 5 | 0.080 (0.045;0.114) | <.0001 |
|  | 15 | 0.002 (-0.013;0.017) | 0.7792 |
|  | 25 | -0.034 (-0.058;-0.010) | 0.0057 |
| Amikacin or vancomycin | 5 | 0.069 (0.036;0.102) | <.0001 |
|  | 23 | 0.007 (-0.007;0.022) | 0.3266 |
|  | 25 | -0.022 (-0.044;0.001) | 0.0610 |
| Amikacin or vancomycin, corrected for ibuprofen | 5 | 0.063 (0.030;0.096) | 0.0002 |
|  | 15 | 0.011 (-0.003;0.026) | 0.1314 |
|  | 25 | -0.013 (-0.035;0.009) | 0.2556 |

**Supplementary Table 3**

Cumulative effect of drug administration on Pottel scores during treatment over consecutive days. CI: confidence interval. P-value for difference with zero consecutive days (i.e. all days without Ibuprofen administration on the previous day).

| **Drug** | **Number consecutive days** | **Mean Pottel score (95% CI)** | **P-value** |
| --- | --- | --- | --- |
| Ibuprofen | 0 | 1.018 (0.994;1.042) |  |
|  | 1 | 1.048 (1.012;1.085) | 0.0393 |
|  | 2 | 1.089 (1.053;1.126) | <.0001 |
|  | 3 | 1.101 (1.063;1.140) | <.0001 |
|  | 4 | 1.109 (1.059;1.160) | <.0001 |
|  | ≥5 | 1.132 (1.079;1.185) | <.0001 |
| Ibuprofen corrected for antibiotics | 0 | 1.020 (0.995;1.044) |  |
|  | 1 | 1.053 (1.016;1.089) | 0.0254 |
|  | 2 | 1.094 (1.057;1.131) | <.0001 |
|  | 3 | 1.106 (1.068;1.145) | <.0001 |
|  | 4 | 1.113 (1.062;1.163) | <.0001 |
|  | ≥5 | 1.135 (1.082;1.187) | <.0001 |
| Amikacin or vancomycin | 0 | 1.027 (1.002;1.052) |  |
|  | 1 | 1.030 (0.995;1.064) | 0.8324 |
|  | 2 | 1.035 (1.000;1.071) | 0.5373 |
|  | 3 | 1.034 (0.997;1.071) | 0.6399 |
|  | 4 | 1.042 (1.001;1.084) | 0.3838 |
|  | ≥5 | 1.055 (1.022;1.088) | 0.0194 |
| Amikacin or vancomycin, corrected for ibuprofen | 0 | 1.024 (0.999;1.048) |  |
|  | 1 | 1.033 (0.999;1.067) | 0.4633 |
|  | 2 | 1.037 (1.002;1.072) | 0.3364 |
|  | 3 | 1.037 (1.001;1.074) | 0.3519 |
|  | 4 | 1.046 (1.005;1.087) | 0.2030 |
|  | ≥5 | 1.060 (1.027;1.093) | 0.0021 |
